# Comparing the Effects on Learning Outcomes of Tablet-Based and Virtual Reality–Based Serious Gaming Modules for Basic Life Support Training: Randomized Trial

**DOI:** 10.2196/13442

**Published:** 2019-05-01

**Authors:** Emin Aksoy

**Affiliations:** 1 Acibadem Mehmet Ali Aydinlar University Center of Advanced Simulation and Education Istanbul Turkey

**Keywords:** serious gaming, virtual reality, health care education

## Abstract

**Background:**

Serious gaming is recognized as a training tool due its potential for a risk-free educational environment. There is still limited research about using serious gaming modules for emergency skills training.

**Objective:**

The aim of this study is to compare the effects on the knowledge level of participants after using a tablet-based serious game and a virtual reality (VR)–based serious game for Basic Life Support using a pretest/posttest method.

**Methods:**

The study was designed as a randomized trial comparing pretest and posttest results. A tablet-based and VR-based serious game with identical content was used for 40 participants. Over half of them (22/40, 55%) were included in the VR group and just under half (18/40, 45%) were in the tablet group. Student *t* test and Wilcoxon signed rank tests were used to determine the relation between the dependent and independent variables. In order to determine the effect size of the results, the effect size calculator (Cohen *d*) for *t* test was used. There is a significant difference between pre- and posttest results in both groups (*P*=.001; Wilcoxon).

**Results:**

Mean posttest results were significantly higher in both groups. The posttest results were significantly higher in the VR group in terms of pre- and posttest changes (*P*=.021; Student *t* test).

**Conclusions:**

Past research studies have shown that serious gaming presents a favorable additional tool for medical education. The results indicate that both serious gaming modules are effective and that VR-based serious gaming is more efficient in terms of learning outcome than tablet-based gaming.

## Introduction

In recent decades, new educational techniques have been adapted to address the constantly changing needs and expectations of educators and learners due to advances in technology. A reason for this shift was that students traditionally must learn the same thing at the same time, with the result that classes and training sessions are frustratingly hard for some and too easy for others [1]. The predominance of interactive learning tools over static reading material exercises has been shown as more effective [2]. It has also been shown that game technologies can positively affect learning performance, with learners quickly mastering and applying new skills and information, thinking laterally and strategically [3]. Experimental studies have largely supported the effectiveness of the game-based learning approach [4,5].

Medical students’ experience and attitudes towards video games were investigated in a recent study with the conclusion that medical students held exceptionally ideal perspectives about the use of serious games [6]. But there still is limited research focused on the use of serious gaming modules for emergency training.

During the initial stages of their training, medical students, nurses, and other health providers are introduced to international Basic Life Support (BLS) algorithms. Traditional courses consist of theoretical lectures followed by hands-on training on a cardiopulmonary resuscitation (CPR) manikin. Considering the increasing use of computer-based education tools in medical education and the worldwide encouragement of teaching BLS Guidelines with video/personal computer (PC) self-learning courses with negligible or no educator training, we aimed to compare the efficacy of a tablet-based module and a virtual reality (VR)­–based serious gaming module on BLS training. Mentors can use the simulated content provided by VR to empower trainees to participate in the learning process [7]. Due to the capacity of VR to attract learners and completely draw them into a virtual world, educators are frequently willing to use VR-based learning modules in learning activities [7]. In addition, the use of VR for training provides a safe learning environment, since it minimizes the physical risks of real-life training while enlarging the scope of learning and expanding trainees’ motivation. The 3DMedSim tablet-based BLS serious gaming app and 3DMedSim VR-based BLS serious gaming modules offer another viewpoint for BLS training and provide trainees dynamic learning by immersing the learner in an environment that reproduces realistic emergency situations. Both systems are compatible with the European Resuscitation Council (ERC) 2015 Algorithm.

The aim of this study is to compare the effect of the knowledge level of participants after using the tablet-based serious game for BLS and the VR-based serious game for BLS with the help of a pretest and a posttest.

## Methods

This study was designed as a randomized trial comparing pretest and posttest results of participants. After approval by the Ethical Committee of Acibadem Mehmet Ali Aydinlar University, 50 first-semester students of Acibadem Mehmet Ali Aydinlar University Vocational School for Paramedics volunteered to participate in this study. The participants (N=50) were randomly divided into two groups with 25 participants each. Since we had some dropouts due to personal reasons, we ended up with 22 of 25 (88%) participants in the VR group and 18 of 25 (72%) in the tablet group using the tablet version of our serious game. The participants were informed about the study and filled out consent forms. None of the participants received any prior education about the ERC 2015 BLS algorithm and had no prior VR experience. The 3DMedSim tablet-based BLS serious gaming app and 3DMedSim VR-based BLS serious gaming module identical content and difficulty level were used for this study. Both serious gaming modules were developed and created by our center, and these modules have been used for training the last 3 years.

Students from both groups had to complete a pretest to assess their prior knowledge on BLS procedures. After the BLS training, students in both groups then had to take a posttest to assess their acquired knowledge. The content of the pre- and posttest is shown in Table 1. The difference between the pre- and posttest was the scrambled order of questions and answers.

Student *t* test and Wilcoxon signed rank test were used to analyze the results in order to determine the relation between the dependent and independent variables. The analysis was performed using MedCalc Statistical Software version 12.7. In order to determine the effective size of the results, the effect size calculator (Cohen *d*) for *t* test was used.

**Figure 1:**
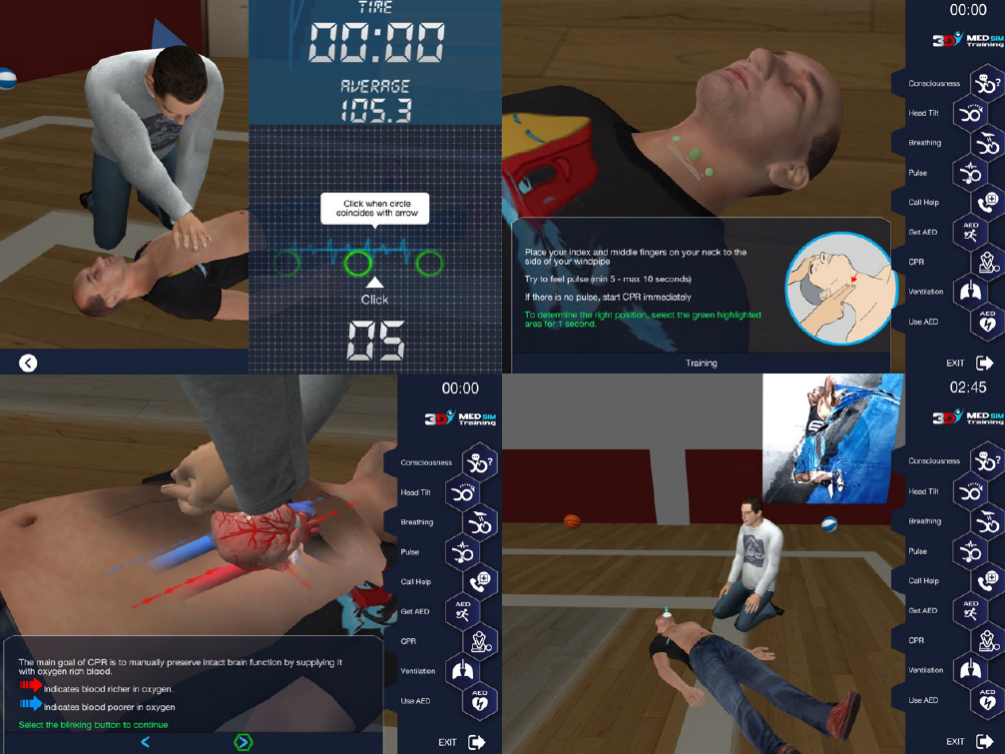
Screen captures from the Adult Basic Life Support Module.

The tablet version of the BLS serious game module is a multilingual serious game app with 3D and interactive features, based on the ERC 2015 Guidelines. The training module guides the user step-by-step through a scenario. The user has to go through a training mode first followed by the self-test mode. When using the serious game module, the user is not in passive reader/learner mode. The user is first instructed about the correct actions to be taken and then expected to interact with the software and “play” the rescuer role interactively. The user was expected to finish one stage correctly before continuing with the next one. The app is implemented as a 3D real-time game allowing the user to interact and see the scene from different angles. A few screen captures from the Adult BLS Module are shown in Figure 1.

Once the user successfully finishes the training module, the user has to complete the self-test module. Unlike the training module, the user is not given hints or instructions to follow and is expected to perform the correct steps with correct timing. Login usernames and passwords were sent to the participants by email, after they had downloaded the serious game from the Web by using iTunes or Google Play. The results of the participants were stored with the help of the learning management system. The participants’ results were downloaded from the learning management system of the serious game module and converted to an MS Excel file.

The VR-based version uses a 3D interface instead of the 2D interface used for the tablet-based version. The educational content, scoring system, and flow of the scenario of the tablet-based and VR-based versions were totally identical, as both versions had to be compatible with the international BLS algorithm, ERC 2015 Basic Life Support Protocol [8]. The only difference was the display type used for serious gaming. Due to hygienic issues, the VR goggle’s inner surface had to be disinfected each time that a new trainee initiated the game with VR. This procedure was not necessary for the tablet-based version. The other difficulty faced by the VR system was that more time was needed to load the game compared with the tablet-based version.

**Table 1:** Content of the multiple-choice test for Basic Life Support (BLS).

Questions			Points
**1. The compression to ventilation ratio for one rescuer is the following:**			10
	a. 30:1		
	b. 15:2		
	c. 30:2		
	d. 15:1		
**2. The 2015 American Heart Association guidelines for cardiopulmonary resuscitation (CPR) recommended BLS sequence is the following:**			10
	a. Airway, Breathing, Check Pulse		
	b. Compressions, Airway, Breathing		
	c. Airway, Breathing, Compressions		
	d. Airway, Check Pulse, Breathing		
**3. The proper steps for operating an automated external defibrillator (AED) are the following:**			10
	a. Turn on the AED, attach AED pads, analyze the rhythm, stand clear, and deliver shock		
	b. Turn on the AED, attach AED pads, shock the patient, and analyze the rhythm		
	c. Check pulse, attach AED pads, analyze rhythm, deliver shock to the patient		
	d. Attach electrode pads, check pulse, shock the patient, and analyze rhythm		
**4. After delivering the shock with AED, what should be the next step?**			10
	a. Check pulse again		
	b. Ventilate only		
	c. Do chest compressions		
	d. Proceed with CPR		
**5. The initial BLS steps for adults are the following:**			10
	a. Assess the individual, call 112 and get AED, check pulse, and start CPR		
	b. Check pulse, give rescue breaths, assess the individual and use AED		
	c. Assess the individual, give two rescue breaths, defibrillate and start CPR		
	d. Assess the individual, start CPR, give two rescue breaths and use AED		
**6. The critical characteristics of high-quality BLS are the following:**			10
	a. Not interrupting		
	b. Starting chest compressions within 10 seconds of recognition of cardiac arrest		
	c. Pushing hard and fast		
	d. All of the above		
**7. The second rescuer has arrived with the AED and turned it on. What should be your next step?**			10
	a. Place the pads over the victim’s clothes		
	b. Wait for advanced care to arrive before continuing use of the AED		
	c. Shock the victim		
	d. Place the pads on the victim’s bare chest		
**8. Which one is the most critical component of CPR?**			10
	a. Airway management		
	b. Rescue breathing		
	c. Chest compressions		
	d. All of the above		
**9. Use a head tilt chin lift to open the airway in an adult victim if you do not suspect a cervical spine injury.**			10
	a. True		
	b. False		
**10. Which artery should you check for pulse in an adult?**			10
	a. Radial artery		
	b. Femoral artery		
	c. Carotid artery		
	d. Brachial artery		

**Figure 2:**
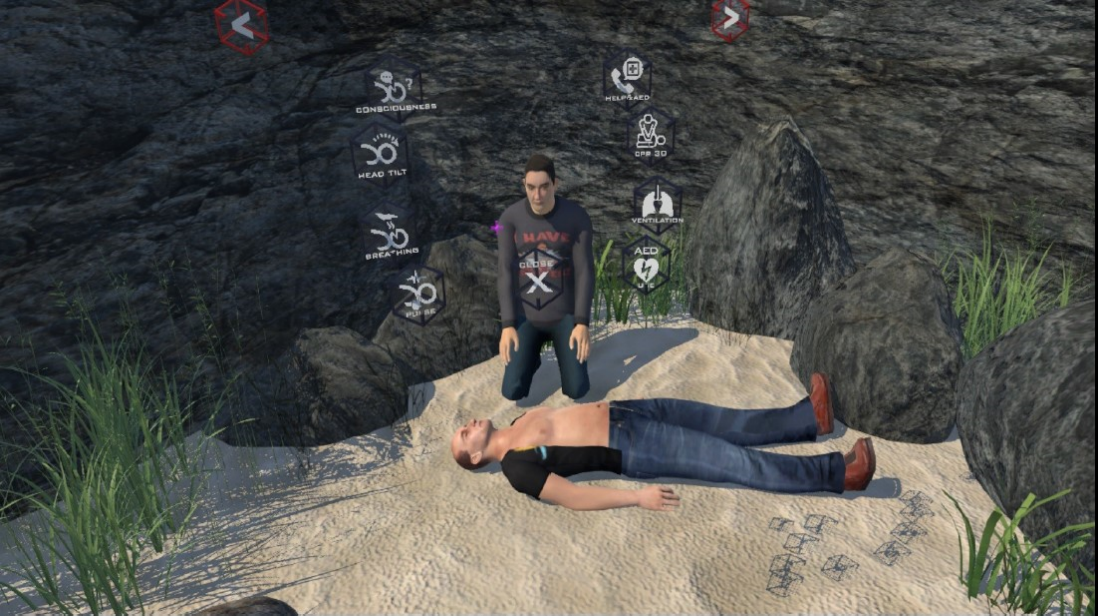
Screen capture from the virtual reality–based serious gaming module.

A very important risk factor of the VR version was the potential problem of dizziness. Similar to motion sickness, VR sickness is caused due to mismatch between the visual and vestibular systems. VR-based serious game scenarios must take this risk into account. Participants were warned about this risk on their written consent forms before using the VR-based version. The VR-based version of the serious game was used in a special room with soft flooring material and special walls covered with soft material in order to minimize the risk of trauma in case of dizziness problems. With the technical improvements of today’s head-mounted systems (HMD) for VR, the risk of dizziness has been minimized due to the higher resolutions and higher refresh rate of the screens used for VR systems compared with the previous generation of similar systems. Unlike the tablet-based BLS module, the VR system is much more immersive. The user can move within the 3D scene freely and after a while feels a part of the scene (Figure 2).

## Results

Pretest results indicated that students in both groups had similar prior knowledge. Statistical analysis was performed using the MedCalc Statistical Software version 12.7.7 [9]. For comparison of two non-normally distributed dependent groups, Wilcoxon signed rank test was used. As seen in Table 2, there is a significant difference between average pre- and posttest results in both groups (*P*=.001). In order to calculate the effect size of the results, Cohen *d* has been used [10]. The effect size of the VR results are significantly higher compared with the tablet version.

**Figure 3:**
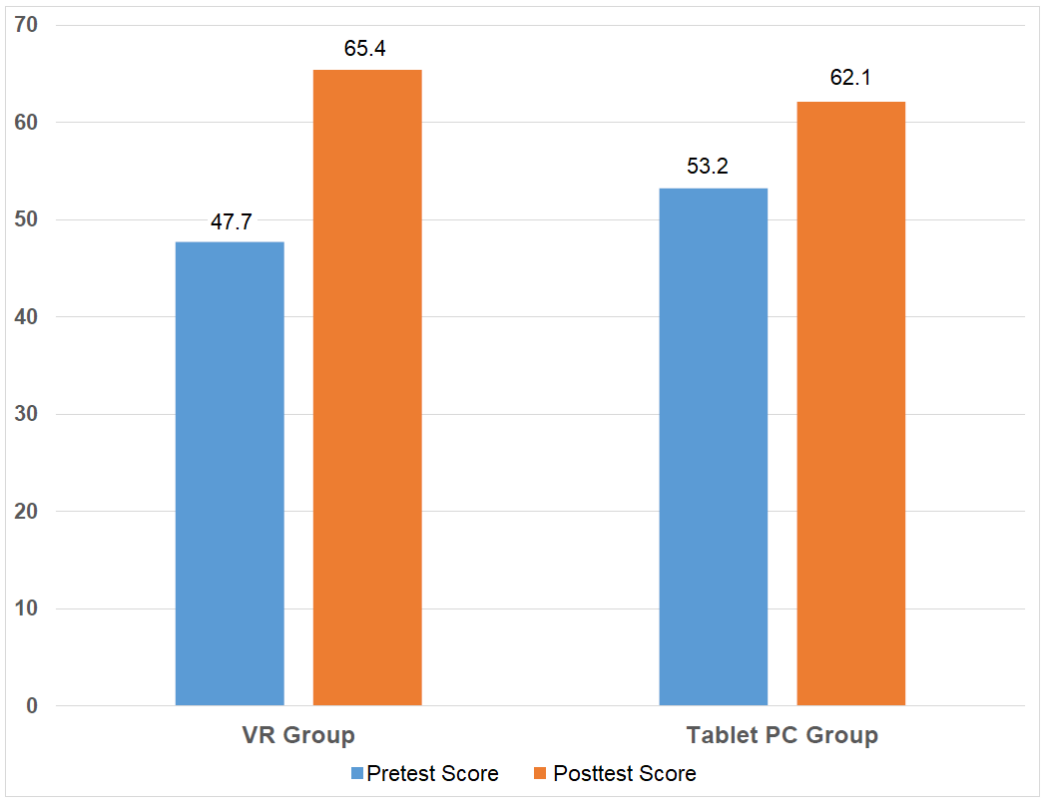
The difference between pre- and posttest results of the tablet (PC) and virtual reality (VR) groups.

Mean posttest results were significantly higher in both groups. The difference between the pre- and posttest results were significantly higher in the VR group, and these data were statistically significant (*P*=.021; Student *t* test) as seen in Table 3 and Figure 3.

**Table 2:** Statistical analysis of pre- and posttest results of the tablet personal computer (PC) and virtual reality (VR) group using Wilcoxon test and effect size calculation results with Cohen *d*.

Group	Pretest		Posttest		*P* value^a^	Cohen *d*
	Mean (SD)	Median (min-max)	Mean (SD)	Median (min-max)		
VR	47.7 (9.5)	46 (37-69)	65.4 (10.6)	62 (46-88)	<.001	1.75
Tablet PC	53.2 (11.3)	54 (31-69)	62.1 (8.5)	62 (46-77)	.001	0.89

^a^Wilcoxon test.

**Table 3:** Difference between the pre- and posttest results of the virtual reality (VR) and tablet personal computer (PC) group.

Variable	VR		Tablet PC			*P* value^a^	
	Mean (SD)	Median (min-max)		Mean (SD)	Median (min-max)		
Difference between pre- and posttest results	–17.6 (11.6)	–16.5 (–42 to 8)		–8.9 (11.1)	–8 (–24 to 16)		.021

^a^Student *t* test (*P*<.05).

## Discussion

### Principal Findings

In various studies, it was shown that the positive impact of active learning methodologies includes increased content understanding, enhanced learning, long-term memory retention, and enhanced collaboration and motivation [11-13]. Serious gaming provides both interaction and motivation for digital learners [1,5]. These technologies are being widely used in different areas, but there are a few studies in medical education [14-18]. Our study also shows us that tablet-based and VR-based serious gaming for the BLS training of health care students are effective in terms of active learning.

Bandura’s Social Learning Theory suggests that individuals learn from each other, by means of perception, impersonation, and demonstration [19]. This theory acts like a bridge between behaviorist and cognitive learning theories because it incorporates attention, memory, and motivation [19]. By using VR-based gaming modules, learners engage with the virtual world and learning material thus providing learners a maximum amount of perception, impersonation, and demonstration.

In our study, we used the 3DMedSim tablet-based BLS serious gaming app and 3DMedSim VR-based BLS serious gaming modules, which are both highly interactive and provide self-directed learning opportunities.

In a study by Fabio Buttussi, the impacts on knowledge acquisition and level of self-efficacy, engagement, and presence by using different kinds of displays were evaluated [20]. The displays used in this study were a standard desktop monitor, a narrow field HMD with 3 Degrees of Freedom (DOF) tracker, and a wide field HMD with 6-DOF tracker. Results demonstrated that the display type had a critical impact on the level of engagement and presence [20].

In our study, we also found that because of immersion effects, VR HMD was more effective compared to the tablet PC. However, we did not evaluate the maintenance of knowledge after a 2-week period.

In the study by Moro et al, both VR and augmented reality (AR) were found to be equally effective for learning anatomical structures as tablet PCs while having the advantage of higher levels of learner immersion and engagement [21]. Trainees’ views of each learning mode and adverse effects experienced were recorded. The differences between in VR, AR, or tablet based versions were found to be insignificant. Adverse effects like headaches, dizziness, and blurred vision were detected in some participants.

In a study by Akshay Vankipuram et al, it was also shown that use of VR-based training modules for medical education could improve the skills of clinicians [22]. There is still limited research about using VR in emergency skills training. Serious gaming was found to be an effective method for active and self-directed learning and for teacher and students’ time constraints by giving the educator and learner flexibility in the learning environment [17]. VR has been perceived for its huge educational potential in risk-free clinical training [23].

In another study, it was mentioned that the use of these technologies has upgraded the adequacy of medical education and training and raised the level of diagnosis and treatment [24]. Another study reveals that VR-based Advanced Cardiac Life Support training can provide a learning experience similar to face-to-face training [14].

Our study supports the importance and positive effect of using serious gaming as a complementary tool in medical education. Table 3 shows that the results favor VR-based serious gaming due to its immersive effect on the participants (*P*=.021 calculated with Student *t* test when *P*<.05). When the effect size was calculated by using Cohen *d* [10], the effect size of the VR results is significantly higher compared with the results of the tablet version (Table 2).

### Limitations

One the limitations in our study was that the participants were not familiar with using VR-based systems in the beginning of the study compared to the group using tablet PCs for the serious gaming app. Due to widespread use of tablets and mobile phones in the Turkish population, participants encountered no difficulties using the tablet-based app. VR-based systems are rather new technologies with very few people having access to this technology at this time in our country. Training on using VR hardware was given to participants before using the VR-based game. Although there was a risk of feeling dizzy while using the VR-based version, this problem was not encountered during this study. Since we used the highest flickering rate and screen resolution with today’s technology, we did not encounter any kind of clinical problems such as dizziness or headache during VR training.

### Conclusions

We contend that VR-based serious gaming provides interactivity and a higher level of presence due to higher immersion levels, therefore it is already being widely used for training purposes in many areas. But there are only a few apps being used in medical education. Despite this, we believe that VR-based serious gaming modules will have many areas of application in medical education as well. Our study indicates that serious gaming has a positive effect on the learning outcome of digital learners. Further studies need to be performed on the effectiveness of serious gaming in health care training.

## Abbreviations

AED: automatic external defibrillator
AR: augmented reality
BLS: Basic Life Support
CPR: cardiopulmonary resuscitation
ERC: European Resuscitation Council
HMD: head-mounted display
PC: personal computer
VR: virtual reality

## Appendix

Multimedia Appendix 1 CONSORT‐EHEALTH checklist (V 1.6.1).
